# A case of acute longus colli tendinitis: an inflammatory disease that causes sudden neck pain and backward neck bending difficulty

**DOI:** 10.1093/omcr/omac009

**Published:** 2022-02-19

**Authors:** Hiroshi Hori, Takahiko Fukuchi, Hitoshi Sugawara

**Affiliations:** Department of Comprehensive Medicine 1, Division of General Medicine, Saitama Medical Center, Jichi Medical University, Saitama, Japan; Department of Comprehensive Medicine 1, Division of General Medicine, Saitama Medical Center, Jichi Medical University, Saitama, Japan

## CASE PRESENTATION

An 83-year-old man visited our hospital with sudden neck pain. He could not rotate or bend his neck backward because of the pain; however, he was conscious and did not report a headache or pain on swallowing. Blood tests revealed a high C-reactive protein level (9.68 mg/dl).

**Figure 1:**
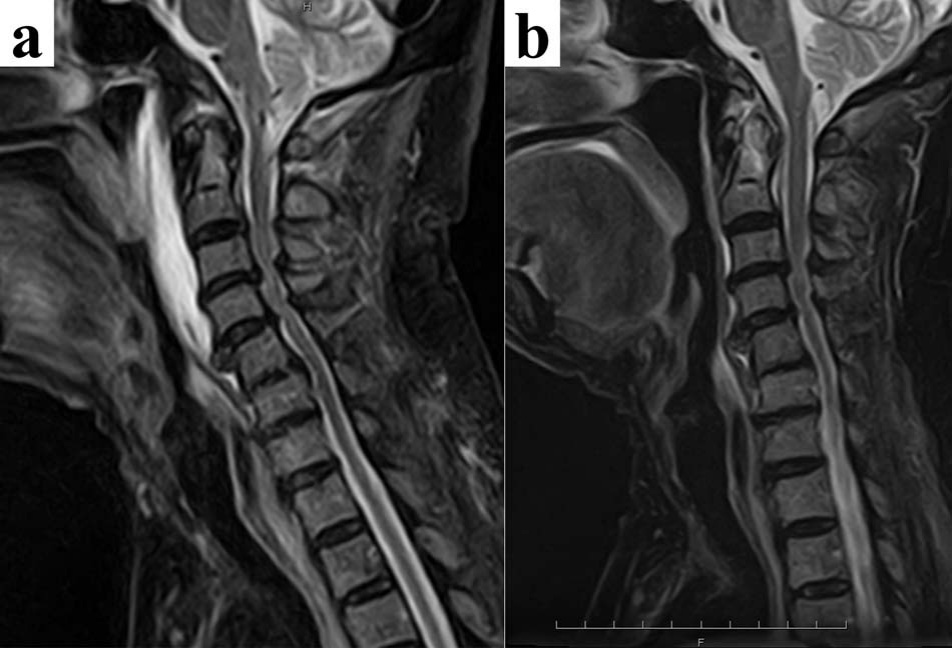
(**a**) A magnetic resonance image shows a strong signal area in front of the cervical vertebral body which coincides with the longus colli muscle in the STIR sequence. (**b**) One week after the administration of loxoprofen, the STIR sequence in the magnetic resonance image shows improvement of the swelling and hyperintensity in the longus colli muscle.

A magnetic resonance imaging (MRI) showed a strong signal in front of the cervical vertebrae which coincided with the longus colli muscle in the short inversion time inversion recovery (STIR) sequence (Fig. 1). There was no indication of cervical vertebrae or intervertebral disk inflammation, and an endoscopy ruled out a retropharyngeal abscess.

We diagnosed the patient with acute longus colli muscle tendinitis and prescribed loxoprofen. The pain improved rapidly, which allowed for cervical rotation and backward neck bending. An MRI 1 week after treatment initiation showed a marked improvement of the longus colli muscle swelling hyperintensity.

The longus colli muscle, which functions as the neck flexor muscle, is located anterior to the first–third thoracic vertebrae. Acute longus colli tendinitis is an inflammatory disease characterized by acute neck pain (94%), limited range of motion (45%) and odynophagia (45%) [1]; however, these symptoms are nonspecific. Acute longus colli tendinitis is relatively rare [2]; therefore, it is underdiagnosed [3]. Misdiagnosis may lead to unnecessary surgical procedures, punctures and antibiotic administration. Non-steroidal anti-inflammatory drugs (NSAIDs) are highly effective, however [3].

Differential diagnoses include retropharyngeal abscess, acute thyroiditis, trauma, spondylitis and meningitis; therefore, diagnostic imaging is important. Longus colli tendon calcification has been confirmed by computed tomography (CT) or MRI in 76% of cases and by anterior soft tissue swelling in 79%. This disease is characterized by diffuse longus colli hyperintensity, as observed through T2 or STIR in an MRI.

If a CT/MRI shows diffuse long neck muscle inflammatory swelling with acute-onset neck pain, longus colli tendinitis should be suspected and NSAIDs should be administered.
